# A case of IgG4-related kidney disease with predominantly unilateral renal atrophy

**DOI:** 10.1007/s13730-018-0355-9

**Published:** 2018-07-30

**Authors:** Akari Takeji, Kazunori Yamada, Dai Inoue, Ichiro Mizushima, Satoshi Hara, Kiyoaki Ito, Hiroshi Fujii, Kenichi Nakajima, Kazuaki Mizutomi, Masakazu Yamagishi, Mitsuhiro Kawano

**Affiliations:** 1 Division of Rheumatology, Kanazawa University Hospital, Kanazawa University Graduate School of Medicine, 13-1, Takara-machi, Kanazawa, Ishikawa 920-8640 Japan; 2 Department of Advanced Research in Community Medicine, Kanazawa University Graduate School of Medical Sciences, Kanazawa, Ishikawa Japan; 3 Department of Radiology, Kanazawa University Graduate School of Medical Science, Kanazawa, Ishikawa Japan; 4 Department of Nuclear Medicine, Kanazawa University Hospital, Kanazawa, Ishikawa Japan; 5 Internal Medicine, Kaga Medical Center, Kaga, Ishikawa Japan; 6 Division of Cardiology, Kanazawa University Graduate School of Medicine, Kanazawa, Ishikawa Japan

**Keywords:** IgG4-related disease, IgG4-related kidney disease, Tubulointerstitial nephritis, Unilateral renal atrophy

## Abstract

A 73-year-old Japanese woman was diagnosed with type 1 autoimmune pancreatitis (AIP) without kidney lesions. She was treated with prednisolone (PSL) 30 mg/day, and her AIP symptoms promptly improved, after which the PSL dose was gradually tapered to 5 mg/day. Her renal function had remained normal (serum creatinine 0.7 mg/dL) until 1 year before the current admission without any imaging abnormalities in the kidney. However, during this past year her renal function gradually declined (serum creatinine 1.1 mg/dL). Follow-up computed tomography incidentally revealed unilateral renal atrophy, which rapidly progressed during the subsequent 10-month period without left kidney atrophy. A diagnosis of IgG4-RKD probably due to TIN was made, and we increased the dose of prednisolone to 30 mg/day. 1 month after administration, multiple low-density lesions on both kidneys were improved slightly but almost all lesions persisted as atrophic scars. Our case suggested that unilateral renal atrophy can develop in patients with IgG4-related tubulointerstitial nephritis without hydronephrosis caused by retroperitoneal fibrosis, and that monitoring the serum creatinine levels is not always sufficient, thereby highlighting the importance of regular imaging monitoring to detect newly developing kidney lesions.

## Introduction

IgG4-related disease (IgG4-RD) is a systemic inflammatory disease characterized by an elevated serum IgG4 level, IgG4-positive plasma cell infiltration, and fibrosis in affected organs, which is becoming increasingly well recognized [1–5]. Patients with IgG4-related kidney disease (IgG4-RKD) reveal various renal abnormalities on imaging studies, such as multiple low-density lesions on contrast-enhanced computed tomography (CE-CT), diffuse bilateral renal swelling, and/or a hypovascular solitary nodule [6, 7], resulting in partial or diffuse renal atrophy in some cases [8, 9]. However, unilateral renal atrophy is very rare except in cases of unilateral hydronephrosis caused by IgG4-related retroperitoneal fibrosis [10, 11]. Here, we describe a patient with IgG4-RKD manifesting predominantly unilateral renal atrophy probably due to IgG4-related tubulointerstitial nephritis (IgG4-related TIN).

## Case report

**Fig. 1:**
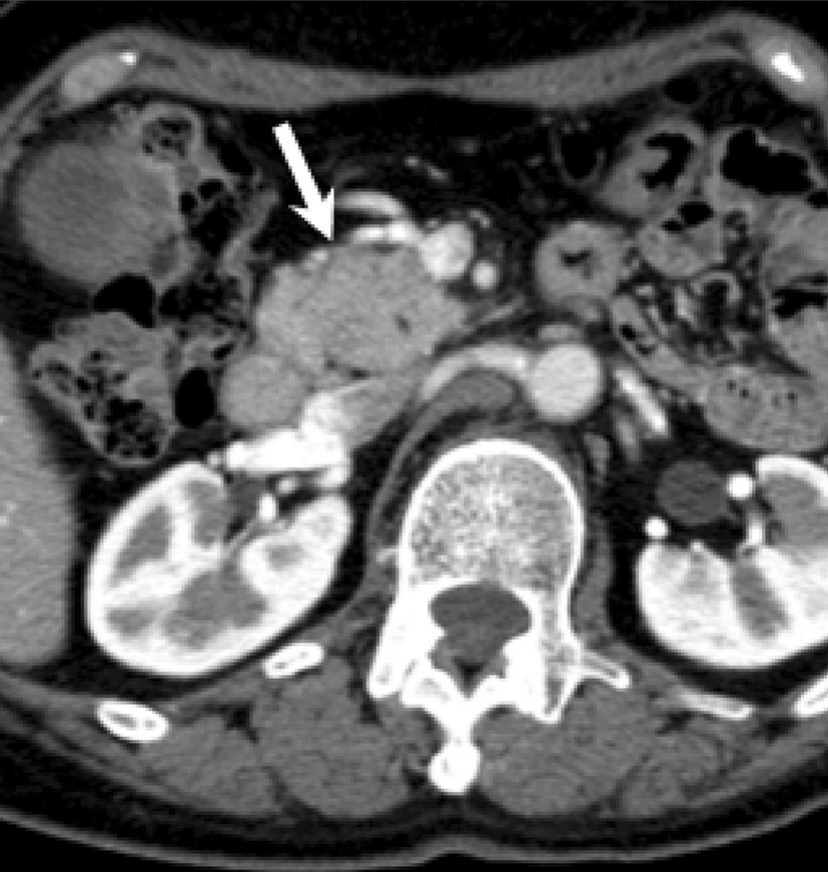
Contrast-enhanced computed tomography (CE-CT) findings at diagnosis of IgG4-related disease. CE-CT revealed a mass in the pancreatic head when she was first referred to our hospital with obstructive jaundice 6 years earlier

**Fig. 2:**
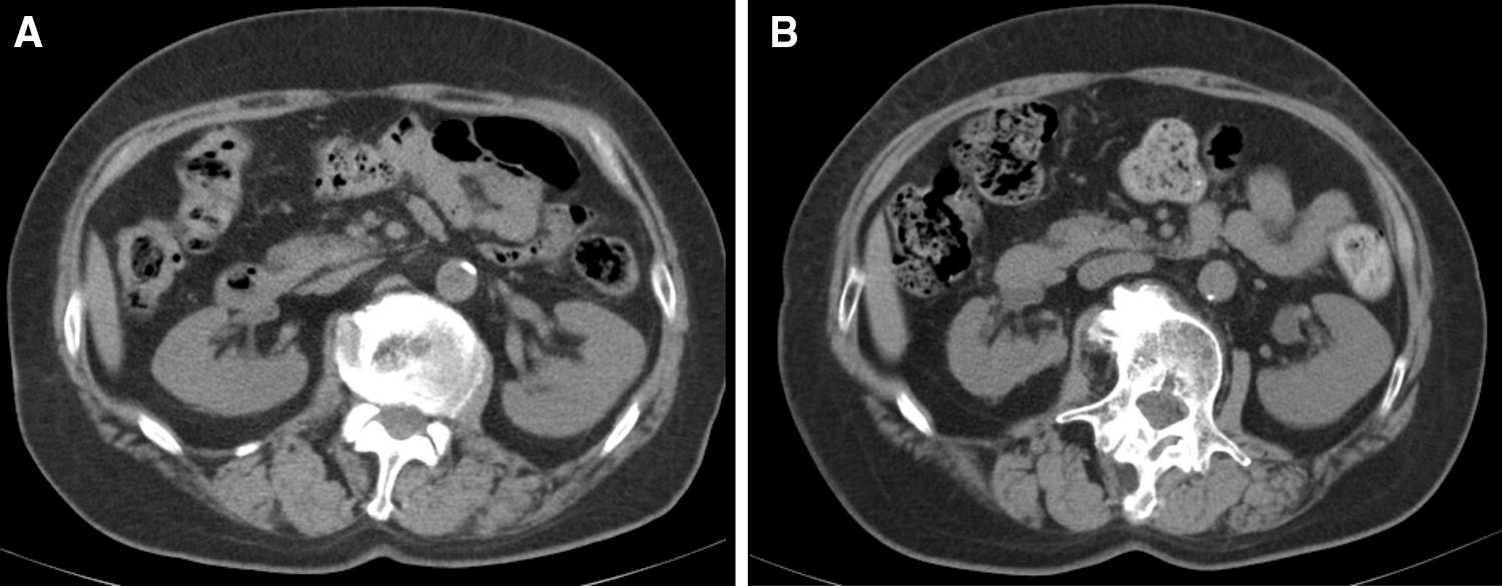
Plain CT images 10 months before (**a**) and on admission (**b**). The right renal parenchymal atrophy showed rapid progression during the 10-month period

**Fig. 3:**
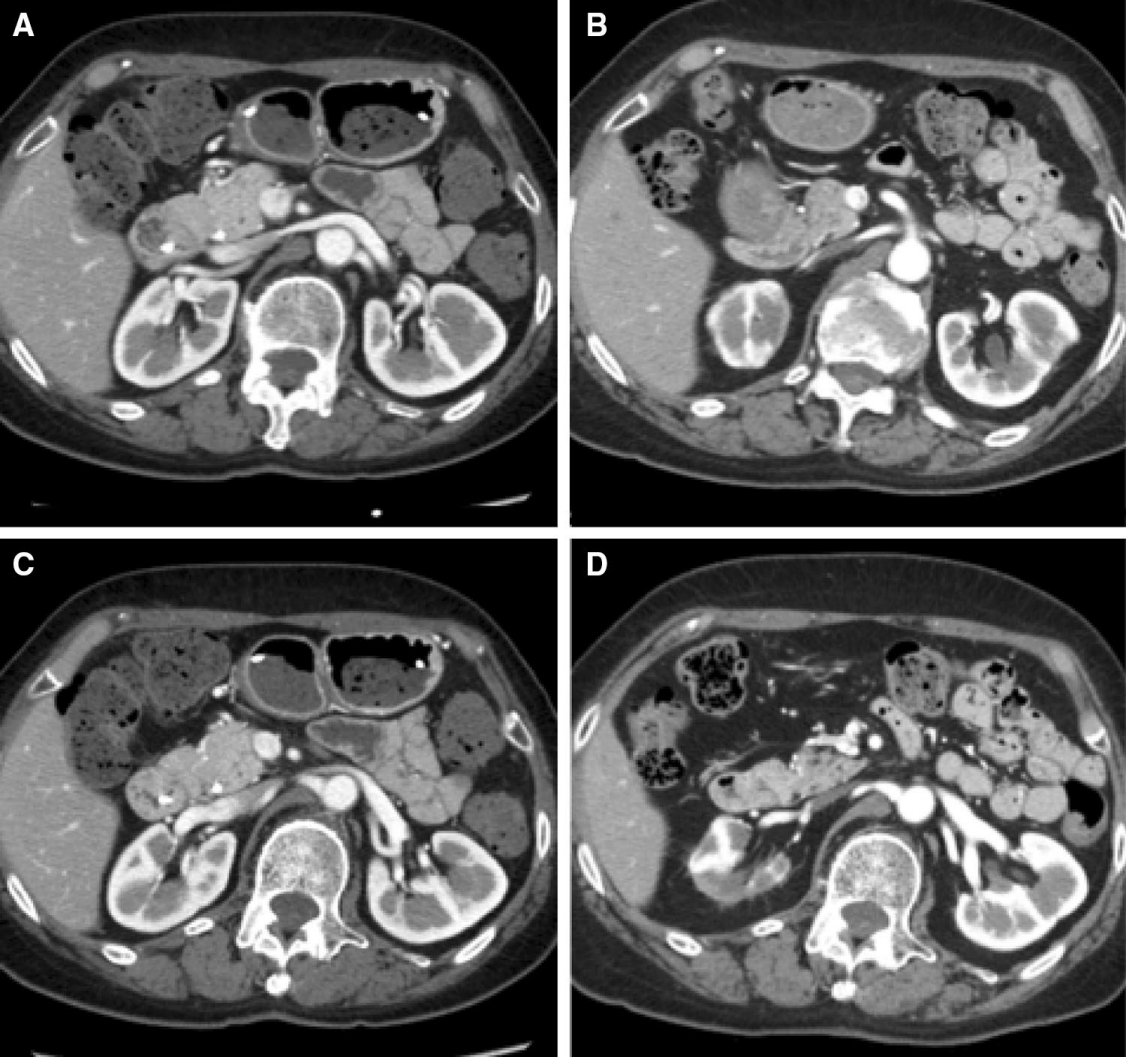
CE-CT images before glucocorticoid therapy for type 1 autoimmune pancreatitis (**a, c**) and on admission (**b, d**). CE-CT on admission revealed multiple low-density lesions in the bilateral kidneys and marked atrophy limited to the right kidney. The left kidney showed only partial atrophy (**b, d**)

**Fig. 4:**
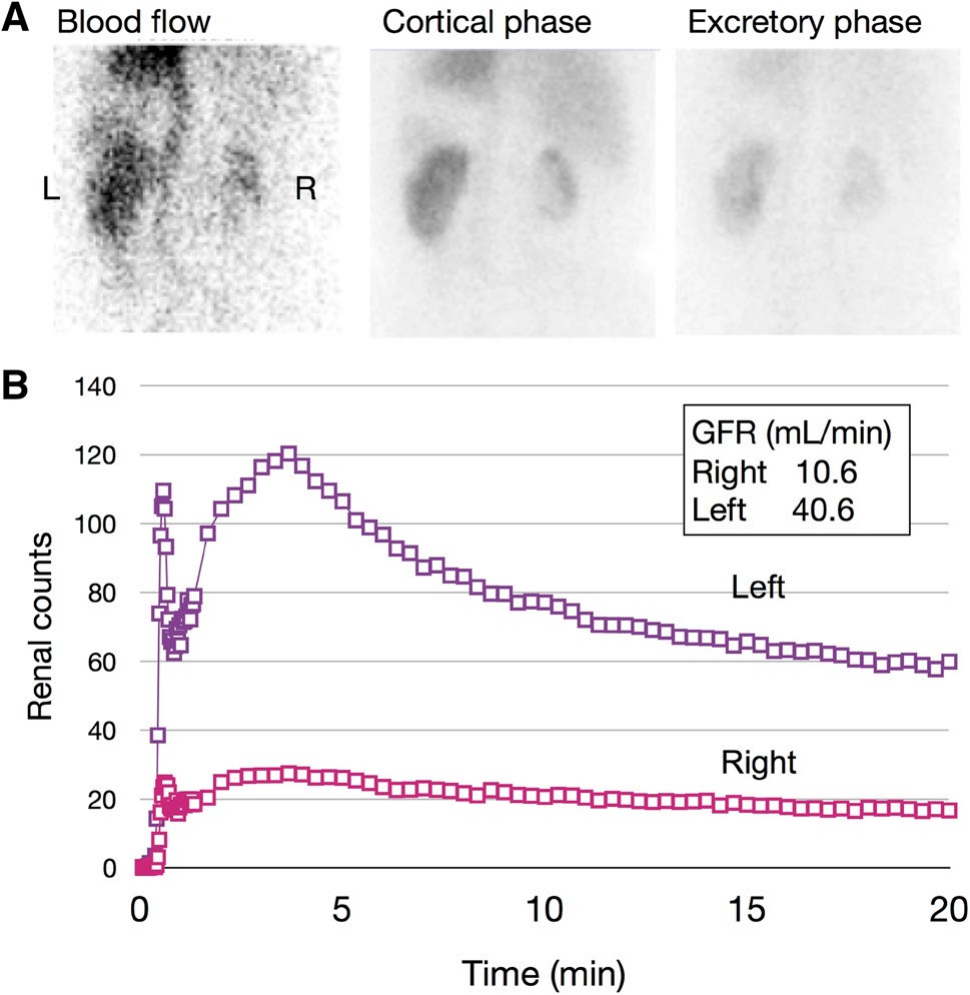
Tc-99m DTPA scintigraphy. Estimated glomerular filtration rate (eGFR) of left kidney was 40.6 mL/min and of right kidney was 10.6 mL/min. Right renal dysfunction was remarkable

A 73-year-old woman was admitted to our hospital for close examination of gradually decreasing renal function. She was first referred to our hospital with obstructive jaundice due to a pancreatic head mass 6 years earlier (Fig. 1). CE-CT showed focal enlargement of the pancreas. Endoscopic retrograde pancreatography showed irregular narrowing of the main pancreatic duct. After closer examinations, type 1 autoimmune pancreatitis (AIP) was highly suspected because she had an elevated serum IgG4 level (378 mg/dL), which exceeded by more than twofold the upper limit of the normal range. She was treated with prednisolone (PSL) 30 mg/day, after which her symptoms promptly improved with serum IgG4 level decreased (165 mg/dL). Finally, a definite diagnosis of type 1 AIP was made based on the Clinical Diagnostic Criteria for Autoimmune Pancreatitis 2011 (level 1 serology and diagnostic steroid trial) [12]. The PSL dose was gradually tapered to 5 mg/day. Before starting treatment, no other characteristic lesions of IgG4-RD commonly found in the kidney or lacrimal and salivary gland were present (Fig. 3a, c). She had a history of hypertension, hyperlipidemia, paroxysmal atrial fibrillation, and old cerebral infarction. Her renal function had remained normal (serum creatinine 0.7 mg/dL) until 1 year before the current admission without any imaging abnormalities in the kidney. However, during this past year her renal function gradually declined (serum creatinine 1.1 mg/dL) and follow-up CT revealed right dominant renal atrophy (Fig. 2). On admission, she was afebrile and her consciousness was clear. On physical examination, blood pressure was 99/54 mmHg and pulse 67 beats per minute. There were no remarkable findings except for slightly swollen bilateral lacrimal glands. Pitting edema on the lower extremities was seen. Laboratory findings (Table 1) included CRP 0.1 mg/dL, creatinine (Cr) 0.88 mg/dL (eGFR 48.1 mL/min/1.73 m^2^), BUN 17 mg/dL, IgG 1261 mg/dL, IgG4 201 mg/dL (IgG4/IgG: 16%), IgA 276 mg/dL, IgM 160 mg/dL, IgE 1025 IU/mL, C3 108 mg/dL, C4 23 mg/dL, and CH50 60 U/mL. Rheumatoid factor, anti-nuclear antibodies, anti-SSA antibodies, anti-myeloperoxidase anti-neutrophil cytoplasmic antibodies (ANCA), and anti-proteinase 3 ANCA were all negative. Neither proteinuria nor hematuria was present. The level of urinary *N*-acetyl-β-d-glucosaminidase (NAG) was 1.9 IU/L and that of urinary β2-microglobulin (β2-MG) was < 75 µg/L. Ga-scintigraphy showed no uptake on kidneys, pancreas, salivary glands, or lymph node. CE-CT on admission demonstrated multiple low-density lesions in the bilateral kidneys that led us consider the possibility of IgG4-RKD. Renal atrophy was seen predominantly in the right kidney (Fig. 3b, d). Renal Technetium-99m diethylene triamine pentaacetic acid (Tc-99m DTPA) scintigraphy revealed marked right renal dysfunction (GFR: left 40.6 mL/min, right 10.6 mL/min; Fig. 4).

**Table 1:** Laboratory data of the present case on admission to our hospital

	Value	Normal range
Urinalysis		
Protein	–	–
Occult blood	–	–
Sugar	–	–
G. cast	–	–
Urinary beta 2 microglobulin (ng/mL)	< 75	
Urinary *N*-acetyl-beta-d-glucosaminidase (IU/L)	1.9	
Blood count		
White blood cells (/µL)	6050	3300–8800
Eo (%)	1.2	0–6
RBC (/µL)	368	430–550
Hb (g/dL)	11.7	13.5–17.0
Plt (/µL)	24.4	13.0–35.0
ESR (mm/h)	47	
Serum chemistry		
BUN (mg/dL)	17	8–22
Cr (mg/dL)	0.88	0.60–1.00
UA (mg/dL)	4.5	3.6–7.0
Na (mEq/L)	146	135–149
K (mEq/L)	4.2	3.5–4.9
Cl (mEq/L)	107	96–108
ALP (IU/L)	144	115–359
γGTP (IU/L)	19	10–47
AST (IU/L)	20	13–33
ALT (IU/L)	15	8–42
LDH (IU/L)	249	119–229
Amy (IU/L)	119	4–113
TP (g/dL)	7.1	6.7–8.3
Alb (g/dL)	3.9	4.0–5.0
HbA1c (%)	6.2	4.3–5.8
FDP-DD (µg/mL)	0.9	< 1.0
Immunological findings		
CRP (mg/dL)	0.1	0.0–0.3
IgG (mg/dL)	1261	870–1700
IgG4 (mg/dL)	201	< 135
IgA (mg/dL)	276	110–410
IgM (mg/dL)	160	33–190
IgE (IU/mL)	1025	< 250
CH50 (U/mL)	60	32–47
C3 (mg/dL)	108	65–135
C4 (mg/dL)	23	13–35
Anti-nuclear antibody	< × 40	–
RF (IU/mL)	4.5	< 20

*RBC* red blood cell, *Hb* hemoglobin, *Plt* platelets, *BUN* blood urea nitrogen, *Cr* creatinine, *UA* uric acid, *ALP* alkaline phosphatase, *γGTP* g-glutamyltransferase, *AST* aspartate aminotransferase, *ALT* alanine aminotransferase, *LDH* lactate dehydrogenase, *CRP* C-reactive protein, *RF* rheumatoid factor

We decided not to perform a renal biopsy because of the right renal atrophy and malformation of the left renal vein in the inferior pole of the left kidney. We performed renal artery ultrasound. Right and left peak systolic velocity (PSV) was 92 and 64 cm/s, respectively, and renal aortic ratio (RAR) was 1.0 and 0.7, respectively. These data supported the absence of renal artery stenosis. d-Dimer levels were not elevated (0.9 µg/mL: normal < 1.0) on admission. Moreover, d-dimer levels were constantly within the normal range throughout the clinical course. Therefore, thrombotic event of the right kidney was also ruled out.

**Fig. 5:**
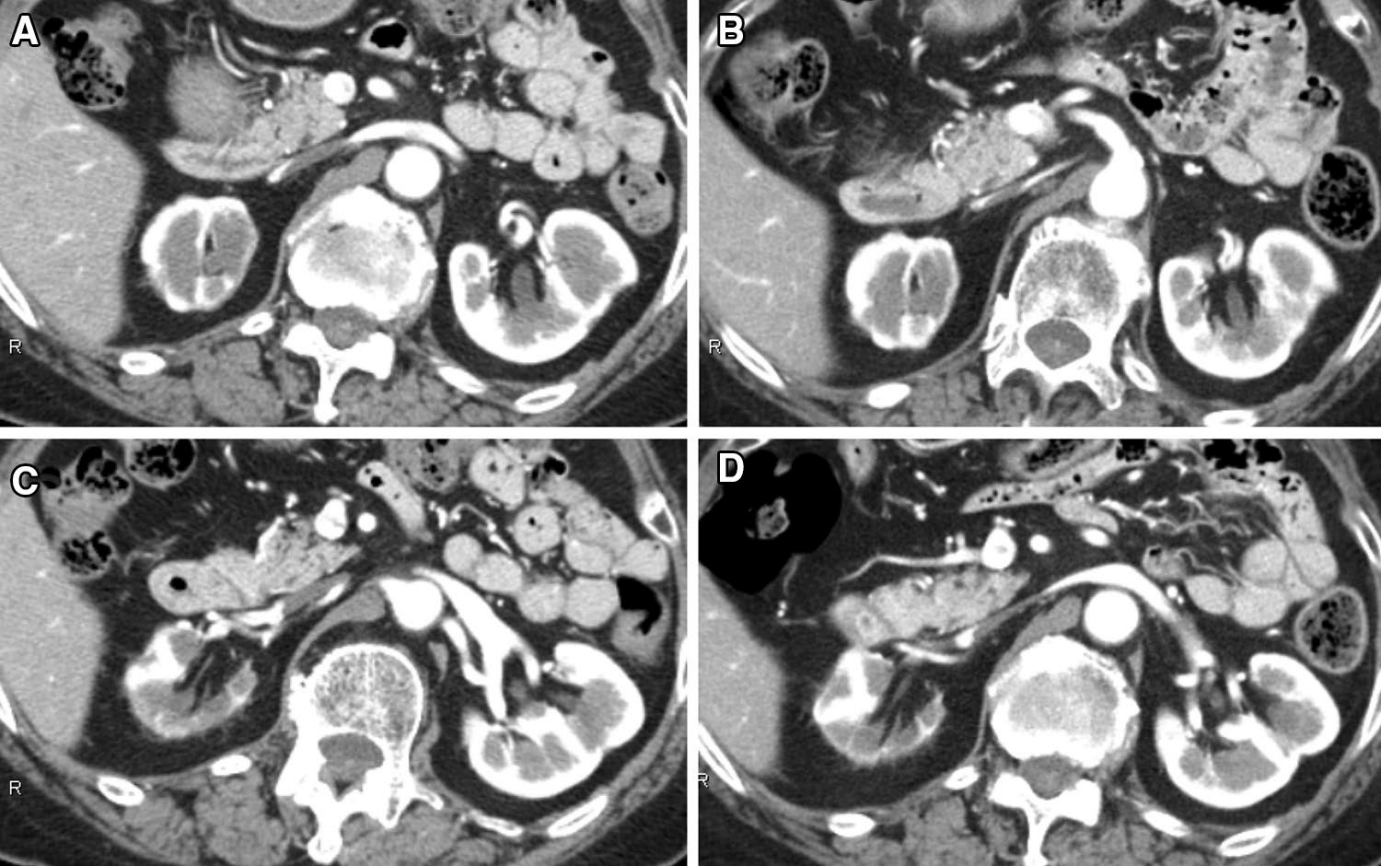
Contrast-enhanced computed tomography images on admission (**a, c**) and 1 month after glucocorticoid therapy (**b, d**). After steroid therapy, the left kidney lesions showing low density and mild partial atrophy demonstrated almost no change

Based on the results of extensive examinations such as chest X-ray, echocardiograph, ultrasound, and gallium scintigraphy, we differentiated IgG4-RKD from other vascular diseases such as renal arterial stenosis, thromboembolism, and aneurysm. Finally, a diagnosis of IgG4-RKD probably due to TIN was made, and we increased the dose of prednisolone to 30 mg/day. 1 month after increasing the dose of corticosteroid, the left kidney lesions showing multiple low-density lesions and mild partial atrophy demonstrated almost no change (Fig. 5).

**Fig. 6:**
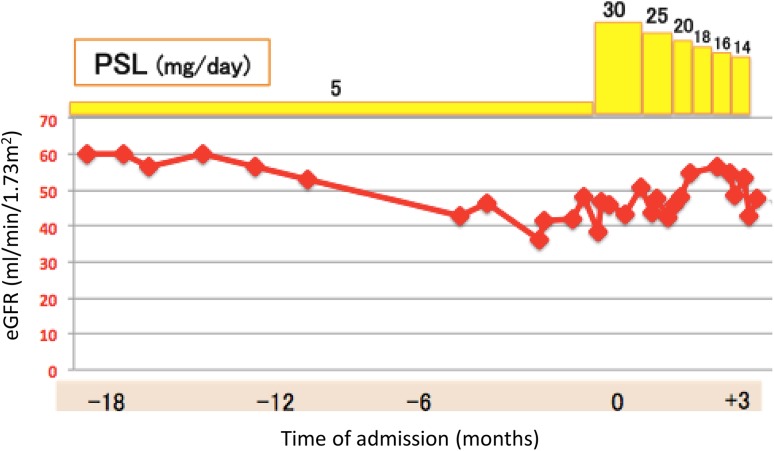
Time course of estimated glomerular filtration rate (eGFR) and prednisolone (PSL) administration. 1 month after increasing the dose of PSL from 5 to 30 mg/day, her renal function improved, and subsequently remained stable. From 3 months after increasing the dose of corticosteroid, the patient has been treated with a maintenance dose of 14 mg/day of prednisolone

3 months after increasing the dose of corticosteroid, the patient was treated with a maintenance dose of 14 mg/day of prednisolone, and renal function was stable (Fig. 6).

## Discussion

We experienced a case of IgG4-RKD with predominantly unilateral renal atrophy without retroperitoneal fibrosis. This case had a very unusual clinical course. At onset, she showed only a pancreatic lesion, with diagnostic imaging not revealing any kidney lesions. Follow-up CT performed during the maintenance therapy did not detect any newly developed renal lesions either until unilateral renal atrophy was detected incidentally and showed rapid progression during the following 10-month period despite the left kidney remaining almost normal.

A characteristic imaging finding of IgG4-RKD is multiple low-density lesions on CE-CT, with almost all cases having bilateral lesions [6, 7]. However, a few reports have rarely described IgG4-RKD with a single mass lesion [13, 14]. In general, rapid unilateral renal atrophy is caused by renal arterial stenosis, renal infarction, thromboembolism, or hydronephrosis associated with periaortitis or inflammatory abdominal aortic aneurysm [15, 16]. Moreover, unilateral renal atrophy could be induced by unilateral hydronephrosis in cases with IgG4-related periaortitis/retroperitoneal fibrosis [10, 11]. However, this is the first reported case with IgG4-RKD to show multiple low-density lesions in the bilateral kidneys on CE-CT and obvious unilateral renal atrophy without retroperitoneal fibrosis or hydronephrosis.

This case highlights the difficulty of knowing when best, or if at all, to start glucocorticoid therapy in patients with IgG4-related TIN [17]. In other words, whether careful watching without therapy is acceptable or not in patients with IgG4-related TIN with preserved renal function is still unanswered. Raissian et al. analyzed the clinical course of 27 patients with IgG4-related TIN with or without glucocorticoid therapy with a mean follow-up time of 14.5 months (range 1–84 months) [18]. In their report, 5 of 27 patients were followed without steroid, of whom two developed elevation of serum creatinine (Cr) levels from around 1.0–1.8 mg/dL while the remaining 3 patients did not despite already showing moderate renal dysfunction. Of 22 of 27 patients receiving corticosteroid therapy, most including those with markedly increased serum Cr levels responded well to the steroid. Saeki et al. reported that all 43 patients with IgG4-related TIN who were treated with steroid achieved stabilization or improvement of renal function, improvement of radiological findings, and resolution of extra-renal manifestations at 1 month after starting therapy [17]. In contrast, Horita et al. described a patient with IgG4-related TIN in whom the morphological findings of multiple low-density lesions dramatically changed into diffuse patchy lesions and the serum creatinine level increased from 0.70 to 1.30 mg/dL during a 5-month interval without corticosteroid therapy [19]. The experience with such reported cases suggests that corticosteroid administration should be immediately initiated in cases with IgG4-related TIN. In our case, the renal function decreased slowly while the renal atrophy progressed rapidly. This implies that careful serum Cr level monitoring is not sufficient to detect rapid renal damage in patients with IgG4-RD as soon as possible, meaning that early treatment would be desirable for IgG4-related TIN patients even if the deterioration rate of renal function is slow.

In our case, urine levels of NAG and β2-MG were not elevated. Mizushima et al. described 6 patients with IgG4-related TIN. In their report, urine β2-MG excretion was not elevated in one patient (16.7%) nor urine NAG level in four (66.7%). Also, urine β2-MG and NAG concentrations fluctuated despite the corticosteroid therapy [20]. Furthermore, Nishi et al. reported elevations of urine NAG and α1-MG levels in 78.6 and 30.8% of the patients, respectively [21]. These findings draw attention to the fact that tubulointerstitial markers are not necessarily elevated in IgG4-related TIN patients.

The present case raises the interesting question of why only the left kidney showed very mild atrophy. In this case, lesions involved the right renal cortex diffusely culminating in severe diffuse atrophy. On the other hand, kidney lesions were limited to only a part of the left cortex, with this being the reason why most of the left kidney was spared severe atrophy (only partial atrophy). We assume that the difference in the degree of atrophy was largely attributable to such difference in the extent of renal involvement (severe in the right, in contrast to only partial in the left).

In conclusion, we experienced a case of IgG4-RKD with predominantly unilateral renal atrophy probably due to newly developed IgG4-related TIN during the clinical course of type 1 AIP with maintenance corticosteroid therapy. The course of this case suggests that monitoring the serum Cr levels is not always sufficient, thereby highlighting the importance of regular imaging monitoring to detect newly developing kidney lesions during the clinical course of IgG4-RD with or without maintenance steroid therapy.
